# Capacity building models for managing multiple long-term conditions in low-and-middle-income countries: a systematic review and gap analysis

**DOI:** 10.1186/s12960-025-00996-3

**Published:** 2025-07-30

**Authors:** Abhinav Sinha, Krushna Chandra Sahoo, Pranab Mahapatra, Sandipana Pati, Jayasingh Kshatri, Srikanta Kanungo, Sandro R. Batista, Bruno P. Nunes, David Weller, Stewart W. Mercer, Sanghamitra Pati

**Affiliations:** 1 ICMR-Regional Medical Research Centre, Bhubaneswar, Odisha India; 2 Department of Psychiatry, Kalinga Institute of Medical Sciences, Bhubaneswar, Odisha India; 3 Bernard Lown Fellow, Harvard TH Chan School of Public Health, Boston, USA; 4 State Institute of Health and Family Welfare, Government of Odisha, Bhubaneswar, India; 5 Faculty of Medicine, Federal University of Goias, Goiania, Brazil; 6 Postgraduate Program in Medical Sciences, Faculty of Medicine, University of Brasilia, Brasilia, Brazil; 7 Postgraduate Program of Nursing, Federal University of Pelotas, Pelotas, Brazil; 8 College of Medicine and Veterinary Medicine, Usher Institute, University of Edinburgh, Edinburgh, UK; 9 South Asian Institute of Health Promotion, Bhubaneswar, India; 10 Department of Health and Kinesiology, University of Illinois Urbana-Champaign, Urbana, IL USA

**Keywords:** Multimorbidity, Patient-centred care, Health communication, Health education, Training, Capacity building, Primary care

## Abstract

**Background:**

The global prevalence of multiple long-term conditions (MLTCs) is increasing, challenging healthcare providers worldwide. In low- and middle-income countries (LMICs), healthcare professionals face additional obstacles in managing MLTCs due to the presence of disease-specific guidelines. This issue is exacerbated by the limited emphasis on both pre-service and in-service training of healthcare professionals on MLTCs within LMICs. Therefore, we conducted a systematic review to synthesize the scientific evidence on training and educational initiatives on MLTCs for health professionals in LMICs.

**Methods:**

We conducted a search across PubMed, Embase, and CINAHL within the domains of ‘multiple long-term conditions’ and capacity-building and systematically reviewed the articles retrieved. The data were synthesized using a healthcare training framework that encompasses objectives, target audience, content and curriculum, training methodology, trainers and facilitators, logistics and implementation, participant engagement and satisfaction, and outcomes. Our findings were reported according to PRISMA guidelines. This systematic review was prospectively registered with the International Prospective Register of Systematic Reviews (CRD42022348483).

**Results:**

Out of 15,981 initial records, 3614 duplicates were removed, leaving 12,367 for title and abstract screening. After full-text review of 204 articles, only four met the inclusion criteria—two from India, one from Ukraine, and one covering multiple African countries (South Africa, Uganda, Ethiopia, and Kenya) demonstrating a scarcity of literature in the field. These studies focused on increasing healthcare providers’ capacity to manage multiple chronic conditions through knowledge, skills, and competency-based training. A ‘train-the-trainer’ approach was emphasized for broader impact in low-income settings. Training methods varied, incorporating interactive sessions and interdisciplinary modular programs. Key recommendations included integrating updated curricula into medical education and addressing logistical barriers. While participants reported improved skills, challenges included sustaining support and adapting programs to local contexts.

**Conclusions:**

MLTC-focused training in LMICs remains limited, with existing programs emphasizing competency-based learning and a ‘train-the-trainer’ approach. Key challenges include sustainability, logistical barriers, and local adaptation. Integrating structured, interdisciplinary training into medical education and professional development, alongside policy support and stakeholder collaboration, is important for future implementation.

**Supplementary Information:**

The online version contains supplementary material available at 10.1186/s12960-025-00996-3.

## Introduction

The increasing burden of multiple long-term conditions (MLTCs) is a major public health challenge globally [1, 2]. Recent systematic reviews indicate the trend of MLTCs is rising in low- and middle-income countries (LMICs), with the prevalence ranging from 0.7 to 81.3% [3]. Moreover, in these countries, the magnitude of non-communicable diseases (NCDs) is compounded by the prevalent chronic infectious diseases (HIV, tuberculosis) leading to a disproportionate burden of MLTCs [4]. MLTCs are associated with poorer patient-reported outcome measures, such as functional ability, psycho-social well-being and health-related quality of life [5–7]. Furthermore, the complex care needs among patients having MLTCs lead to polypharmacy, and increased healthcare utilization and expenditure [8, 9]. Recent studies indicate MLTCs to be associated with a higher risk of frailty, falls and abuse, especially among older adults [10–12].

In LMICs, MLTCs are exacerbated by factors such as gender  inequalities and social deprivation that further limit the ability to seek a continuum of care [13, 14]. In addition, overwhelmed health systems often restrict longer physician consultations, decrease healthcare access, and lead to a rise in physicians’ stress [15]. This is further compounded by the disease-specific guidelines that may be unsuitable for patients with MLTCs [16]. In high-income countries (HICs), recent recommendations such as the National Institute for Health and Care Excellence (NICE) guidelines on managing MLTCs [17] highlight the need for patient-centred approaches and the need for good communication with patients as well as other health professionals in a well-coordinated care model [17]. This also aids in the shared decision-making process that is beneficial for both patients and clinicians.

The increasing number of individuals with MLTCs along with a limited workforce to manage them has been a concern in LMICs [18]. In this scenario, inadequately trained healthcare professionals including community workers, nurses, physicians and otherhealth professionals may aggravate the hardships. A well-trained and motivated workforce is critical to provide optimized patient outcomes [19]. However, hospital-centric medical education results in the training of medical, nursing and other health professionals in specialist clinical environments with limited focus on interdisciplinary training [20]. Nonetheless, the effectiveness of the holistic care approach is based on harmony and synchrony across several healthcare professions that highlight the need for inter-professional care. The health professionals should be familiarized with these newer dimensions from the beginning which could be done by incorporating these concepts into the undergraduate curriculum [21].

While MLTCs pose significant clinical challenges, the primary gap lies in the lack of structured training models for healthcare professionals in LMICs. Existing education systems often emphasize disease-specific management rather than interdisciplinary and patient-centred approaches. To strengthen capacity building, it is essential to examine known education and training models in health professional education. Understanding these frameworks will provide valuable insights integrating MLTCs into existing curricula and designing effective training strategies for LMICs.

In the absence of an integrated management protocol, clinical decision-making for MLTCs is often complicated by conflicting disease-specific guidelines leading to fragmented care-seeking pathways, decreased patient care and poor primary–secondary care interface [22]. To address the varied healthcare challenges of MLTCs, a comprehensive health systems response is essential. Primary healthcare being the first point of contact for most of these patients should be centre for providing continuous and coordinated care [23]. At the same time, there is a need for health systems to strengthen and build health professionals’ competency including MLTCs in the existing educational programs and curricula which are largely lacking in LMICs. Thus, it is relevant to explore and understand the current evidence that can form a base for future policies for providing training on MLTCs in LMICs. Therefore , we conducted a systematic review to synthesize the scientific evidence on training and educational initiatives on MLTCs for health professionals in LMICs.

## Methods

### Protocol and standards

We prospectively registered this systematic review with the International Prospective Register of Systematic Reviews (Registration ID: CRD42022348483) [24]. This systematic review was performed and reported following the Preferred Reporting Items for Systematic Reviews and Meta-analysis (PRISMA) guidelines (Supplementary file 1: Table S1) [25].

### Information sources and search strategy

We performed a comprehensive search using both medical literature databases and grey literature. We searched the electronic databases: Medline (PubMed), Embase, and CINAHL. In addition, the reference list of the included studies was hand-searched.

We included two major concepts: multimorbidity and capacity building for developing the basic search syntax in PubMed. Medical Subject Headings (MeSH) terms for both “multiple long-term conditions” and “capacity building” were used along with various keywords, such as ‘multiple chronic conditions’, ‘multi condition’, ‘polymorbid’ for multimorbidity and ‘module’, ‘guidelines’, and ‘curriculum’ for capacity building. The MeSH term and keywords for each concept were joined with ‘OR’, whereas the concepts were finally added with the ‘AND’ Boolean operator. We developed specific search strategies for each of the databases. Emtree terms were used in Embase along with other specific keywords. The detailed search strategy for each of the databases is provided in Supplementary file 1: Table S2. We included articles published in the English language up to November 2021.

### Eligibility criteria

We included peer-reviewed original studies reporting training programmes addressing the management of MLTCs, and materials on building the capacity of health professionals on MLTCs in LMICs. We also included articles which documented educational programs, course modules, curricula, care protocol, standard treatment protocol, and evidence-based guidelines on MLTCs among LMICs.

We excluded studies that did not focus on training or capacity building of health professionals in managing MLTCs in LMICs. In addition, we excluded commentaries, editorials, newsletters, and any materials that did not provide original research on educational programs, course modules, curricula, care protocols, standard treatment protocols, or evidence-based guidelines related to MLTCs.

### Study selection, data extraction and synthesis

The articles were retrieved from all three databases in ris. format. These studies were then merged after removing duplicates using Endnote software (EndNote × 8.2, London). We used Rayyan (https://www.rayyan.ai/), web-based free software to screen the articles for potential eligibility. In the first phase of screening, two independent reviewers (AS, KCS) assessed the articles based on titles and abstracts. The articles were categorized as relevant, irrelevant or unsure. Articles which were grouped as irrelevant by both reviewers were eliminated, while the unsure studies or articles with a conflict between the two reviewers (one reviewer considered the article as relevant while the other thought it as irrelevant) were further reviewed by a third reviewer (PM). For secondary or full-text review, potentially eligible articles from primary screening were screened by two independent reviewers (AS, KCS) based on inclusion and exclusion criteria. Any differences during the screening of full texts were resolved by a third reviewer (SP2).

Data from included studies were extracted in a preformed and pre-piloted data extraction sheet specially designed for this study. The data extraction sheet comprised author information, year of publication, study design, study setting, country, participants, and capacity-building methods. Data were extracted by two independent reviewers (AS, KCS) and checked for differences by another author (SP1). Any disagreement on the data extraction spreadsheet was resolved by the entire team in consensus. We contacted the authors of the included studies through the email if any data was found to be missing or incomplete. We synthesised and summarised the data using qualitative research methods [26]. A narrative synthesis of the included studies was presented.

The findings are organised based on a comprehensive review of healthcare training programmes, with an emphasis on programme objectives, target audience, content and curriculum, training methodology, trainers and facilitators, logistics and implementation, participant engagement and satisfaction, outcomes and impact, challenges and limitations, and recommendations for improvement.

### Quality assessment

The quality of selected studies was assessed using the Mixed Method Appraisal Tool (MMAT), which is designed for the appraisal of the methodological quality of systematic mixed-method reviews including quantitative, qualitative and mixed-method studies [27]. The tool comprises five screening questions, and 25 criteria for the critical appraisal under five categories: qualitative research (five items), randomized controlled trials (five items), non-randomized studies (five items), quantitative descriptive studies (five items), and mixed method studies (five items) that can be used depending on the study design included. For the quality appraisal we used questions for mixed methods studies, and non-randomized trials (detailed questions in Table 1). Two independent reviewers assessed the risk of bias for all individual studies. Any disagreement between the two reviewers was solved after consulting a third reviewer.

**Table 1:** Risk-of-bias assessment in individual studies

Quantitative non-randomized					
Author, years	Are the participants representative of the target population?	Are measurements appropriate regarding both the outcome and intervention (or exposure)?	Are there complete outcome data?	Are the confounders accounted for in the design and analysis?	During the study period, is the intervention administered (or exposure occurred) as intended?
Millar HL et al., 2015 [28]	Yes	Yes	Yes	Can’t tell	Yes
Bhalla S et al., 2018 [30]	Yes	Can’t tell	Can’t tell	Can’t tell	Can’t tell

Mixed-methods study					
Author, years	Is there an adequate rationale for using a mixed methods design to address the research question?	Are the different components of the study effectively integrated to answer the research question?	Are the outputs of the integration of qualitative and quantitative components adequately interpreted?	Are divergences and inconsistencies between quantitative and qualitative results adequately addressed?	Do the different components of the study adhere to the quality criteria of each tradition of the methods involved?
Lawson C et al., 2017 [29]	Yes	Yes	Yes	Yes	Yes
Laatikainen T et al., 2021 [31]	Yes	Yes	Yes	Can’t tell	Yes

Three studies [28, 29, 31] had lower risk of bias, while one of the study [30] reported a high risk of bias (Table 1).

## Results

**Fig. 1:**
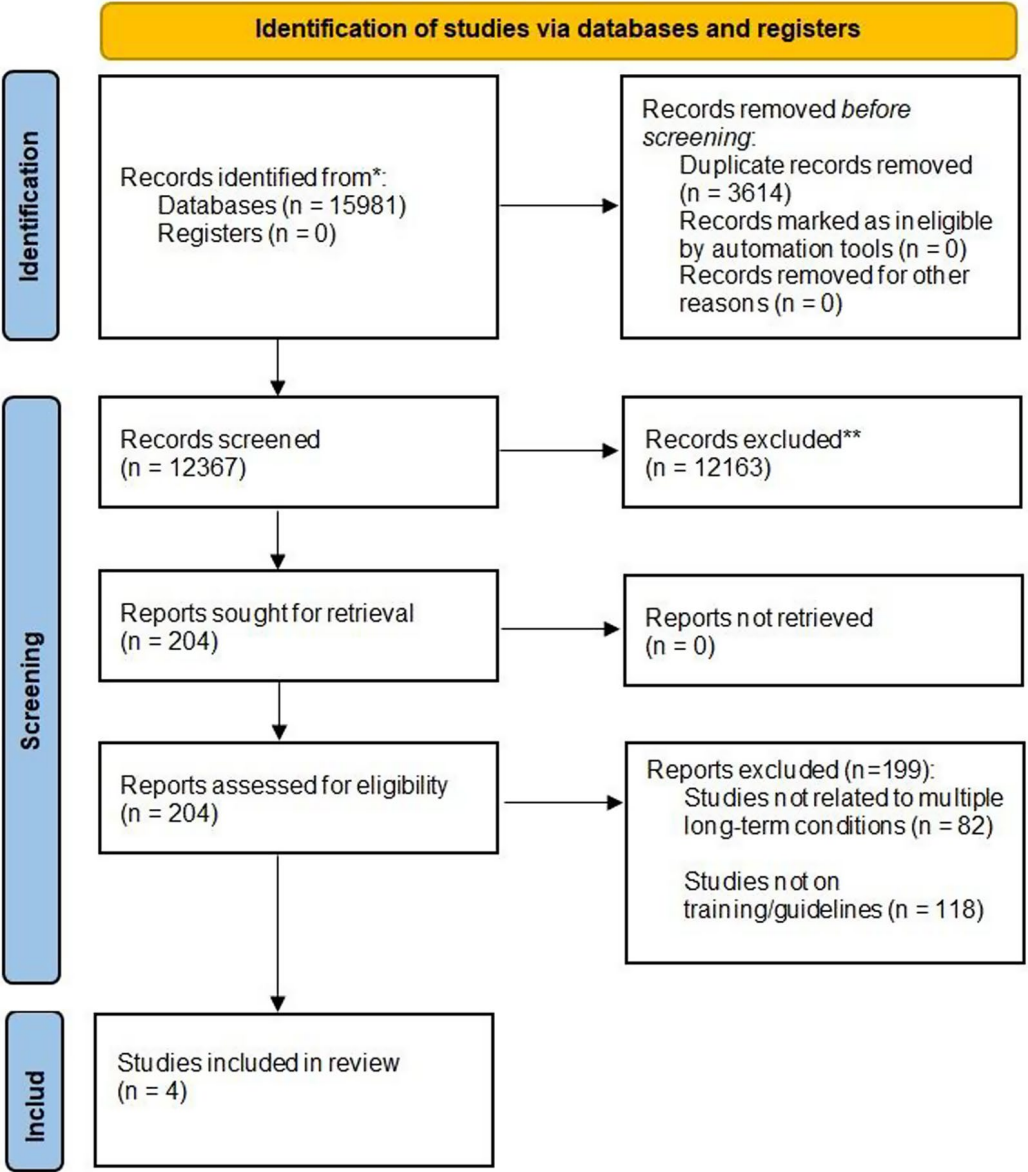
PRISMA flow diagram

We retrieved 15,981 records from all databases, out of which 3614 duplicates were removed, thus selecting 12,367 records for primary screening based on the title and abstract. Following primary screening, we selected 204 articles to be potentially eligible for full-text review out of which four articles [28–31] met the eligibility criteria and were included in the review. We excluded 200 studies during full-text screening out of which 82 were not related to MLTCs. These were included during primary screening as the reviewers were not clear from abstract whether they focus on MLTCs or not. The detailed selection criterion is shown in Fig. 1.

### Characteristics of the included studies

We identified four studies: two from India [29, 30], one from Ukraine [31], and one multi-centric study conducted in four African countries—South Africa, Uganda, Ethiopia, and Kenya [28] which shows a scarcity of literature on the subject. Three studies used the capacity-building model [28, 30, 31], and one study created a framework that could be used in existing curricula [29]. Two of the three studies that tested the implementation of a training module used a non-randomized controlled trial design [28, 29], with one using a pre–post design and the other evaluating the training only after it was completed [30]. Laatikainen et al. used a mixed-method study design, collecting quantitative data through a non-randomized controlled trial and qualitative data through focus group discussions, as well as observing organisational changes in clinics after training [31]. The majority of the studies did not explicitly use the term ‘multimorbidity’, but instead focused on two or more chronic conditions, including the following combinations: diabetes and depression; chronic obstructive pulmonary disease and asthma; and hypertension and diabetes. One study used the term comorbidity, but the framework did not specify an index disease and could be used for multiple long-term conditions [29]. The training ranged from 2 days to 8 months [28–31]. The detailed characteristics of the included studies are provided in Table 2.

**Table 2:** Characteristics of the included studies

Author, years	Country	Study Design	Participants	Chronic conditions considered	Mode of Training	Number of Participants	Intervention	Comparison	Outcomes
Millar HL et al., 2015 [28]	South Africa Uganda Botswana Swaziland Ethiopia Kenya	Uncontrolled, post–post study evaluation using a Likert scale and open-ended reflection on each module and overall workshop	Nurse Practitioners and Nurse educators	Diabetes and Depression	Interactive and participatory teaching and learning approach	175	Ten educational modules Role-play Case-studies Interviewing and assessment Self-management Train-the-trainer guidance	None	Outcomes of each module and items related to teaching methods
Lawson C et al., 2017 [29]	India	Mixed-method, development of framework	Nurse Doctor Pharmacist	Multiple long-term conditions	Teaching through integration in existing curricula across different disciplines (medicine, nursing, pharmacy and other allied health courses)	NA	Integrated Comorbidity Education Framework	Not applicable	Not applicable
Bhalla S et al., 2018 [30]	India	Uncontrolled, pre–post study	Primary Care Physicians	Chronic Obstructive Pulmonary Disease and Asthma	Eight-month modular programme	500	Evidence-based curriculum Case studies	None	Assessment of knowledge of participants
Laatikainen T et al., 2021 [31]	Ukraine	Mixed-method	Primary Healthcare Professionals	Integrated management of Hypertension and Diabetes	Two day course	10,804	WHO package of Essential Non-communicable disease interventions for integrated management of hypertension and diabetes Health education and counselling on healthy behaviours Clinical decision support tools	Yes, Regular practice	Process indicators such as documentation of smoking status, BMI (Body Mass Index), monitoring of blood pressure Outcome indicators: blood pressure and Total cholesterol at normal range

### Program objectives and target audience

This review showed that the included studies [28–31] aimed to improve healthcare providers’ ability to manage multiple chronic conditions through improved knowledge, skills, and core competencies. Lawson et al. introduced an International Comorbidity Education Framework involving diverse professionals [29]. They advocated for training nurses in low-income countries and a ‘train the trainer’ approach for widespread benefits. Millar et al. tailored content to African contexts, integrating WHO principles for non-communicable diseases [28]. Training methods varied, with interactive sessions and interdisciplinary modular programs. Recommendations included integrating new curricula into medical faculties and addressing logistical challenges. Participant feedback was positive, highlighting acquired skills, yet challenges included on-going support and cultural adaptation. Millar et al. emphasized the importance of training nurses in LMICs, recognizing them as the primary healthcare workforce responsible for bridging the gap between patients and their careers [28]. They specifically advocated for a ‘train the trainer’ methodology, which is relevant for empowering nurses to spread the program’s benefits across the country, particularly in African countries.

### Content and curriculum

Each study addressed the perceived needs of trainees to tailor effectiveness of program content. Millar et al. took into account African geopolitical, economic, and social factors when designing their course content, particularly considering how the diagnosis and treatment of conditions such as diabetes and depression should be culturally relevant [28]. Their approach involved a baseline assessment covering evidence-based modules, narratives, fact sheets, and tools to assess depression, alongside tackling the issue of mental health stigma, culminating in the development of actionable plans for nurses’ clinical practices.

Moreover, Lawson et al. started by surveying students’ existing knowledge, evaluating current healthcare curricula, and subsequently elaborating a new content-based education framework centred around six components, or "six Cs": conditions, context, corroboration, conflicts, communication, and collaboration [29]. These elements are complemented by trigger questions that serve as memory aids. Similarly, Laatikainen et al. adhered to the World Health Organization’s Package of Essential Non-communicable Diseases (WHO PEN) principles, focusing on the integrated management of conditions, such as hypertension and diabetes [31]. They focused on key elements such as health education and counselling on healthy behaviours tailored for Ukraine, incorporating clinical decision support tools and measures to enhance efficacy. However, Bhalla et al. detailed a course pedagogy developed through extensive stakeholder interactions across diverse fields associated with medical education and healthcare delivery systems in India [30]. Their curriculum, based on evidence, considers recent advances including diagnostic tests and inhaler device techniques. It incorporates case studies and ensures a thorough evaluation of acquired knowledge.

### Training methodology and trainers

Millar et al. facilitated interactive sessions with a strong emphasis on participatory learning approaches, employing techniques, such as role-playing, interviewing and assessment, self-management strategies, and guidance for trainers to implement their program effectively [28]. Bhalla et al. designed an interdisciplinary, 8-month modular program that was conducted on the job [30], while Laatikainen et al. offered a 2-day face-to-face course [31]. In contrast, Lawson et al. proposed integrating their framework into existing undergraduate and postgraduate curricula [29].

Lawson et al. recommended the adoption of their newly developed curricula by existing medical faculties [29]. Millar et al. established a steering group comprising multidisciplinary representatives, who collaborated in both designing and implementing the course with the support of the International Council of Nurses and other international experts [28]. Bhalla et al. stressed the importance of standardized teaching methods, with their course being supported by national experts and delivered by pulmonologists across various centres in India [30]. To ensure delivery quality, a robust onsite and offsite monitoring and evaluation mechanism was in place, with healthcare and public health professionals serving as observers. Laatikainen et al. delivered courses through regional trainers identified at various sites [31].

### Logistics and implementation focusing on participant engagement, satisfaction and outcomes

The programs varied in duration from 2 days to approximately 8 months, accommodating the schedules of working professionals [28–31]. Careful consideration was given to the selection of the target audience, who were perceived as key ambassadors of the course and potential trainers and implementers in the future [28]. All studies ensured that participants received resource materials, including case studies and tools for future reference [28–31].

Feedback from participants indicated high satisfaction with the course contents, with many expressing that they had acquired new skills beneficial to their routine clinical practice. Millar et al. reported a high positive mean score of approximately 4.69 out of 5 from participants [28]. Qualitative assessments revealed that trainees found the workshops to be "eye-opening." Bhalla et al.’s program achieved considerable success, being adopted by various state governments in India and endorsed by the International Primary Care Respiratory Group for 5 years, with plans for pilot programs in several other countries [30]. Laatikainen et al. observed a significant improvement in knowledge and skills related to the screening and management of non-communicable diseases (NCDs) following the training [31].

### Challenges and recommendations for improvement

Millar et al. noted that nurses often receive training focused on either system-based or disease-based models, which may leave them ill-prepared to manage multiple long-term conditions effectively [28]. In resource-constrained settings, careful selection of participants for such training is important, as they serve as future change agents, regional trainers, and advocates. Studies highlighted the importance of providing a diverse array of modules to meet the perceived needs of participants in capacity-building initiatives. However, Laatikainen et al. found that implementing workshop learning was generally low, as a single training session was insufficient. Lack of managerial support and competing demands further hindered participants from integrating new practices into their clinics effectively [31].

Feedback received by Millar et al. resonated with their team, with major recommendations including providing ongoing support and monitoring of actions, as well as refining workshop materials regarding module flow and time allocation [28]. Lawson et al. suggested cultural adaptation and testing of their framework across various disciplines before implementation [29]. Participants from Ukraine (Laatikainen et al.) emphasized the importance of continued medical education to aid in the successful implementation of new practices in their clinical settings [31].

## Discussion

The review described the healthcare training programs geared towards enhancing healthcare providers’ capabilities in managing multiple long-term conditions. These programs, spanning various geographic regions, shared a common objective of reinforcing knowledge, skills, and core competencies of healthcare professionals for managing MLTCs. This review explores capacity-building programs, examining their objectives, target audiences, content, methodology, facilitators, logistics, engagement, outcomes, challenges, and recommendations for improvement. We seek to gain insights into the strengths, gap, and overall impact of these programs in meeting the needs of healthcare professionals to manage MLTCs.

The programs employed diverse approaches in content design, training methodology, and target audience selection. Participatory learning techniques and culturally relevant content emerged as effective strategies. High participant satisfaction levels suggest that these programs effectively addressed perceived needs and transferred valuable skills applicable to clinical practice. Furthermore, several programs demonstrated significant improvements in participants’ knowledge and skills related to chronic disease management post-training. Nonetheless, there was a paucity of literature on capacity building of healthcare staff for MLTCs in LMICs. Our findings are similar to a systematic review that observed only two studies implemented and evaluated education and training formats for postgraduate medical doctors in the management of multimorbidity in primary/secondary care [32].

Furthermore, the included studies observed that despite the training program being a success, challenges such as limited workshop implementation and inadequate managerial support were identified, signalling the need for ongoing support and monitoring mechanisms. This review highlights the importance of tailored training initiatives in meeting the complex needs of healthcare providers managing multiple chronic conditions. Incorporating feedback and improvement recommendations can improve future programs’ effectiveness in preparing healthcare professionals to deliver comprehensive care across diverse clinical settings. Moreover, decision makers in LMICs can also evaluate and try to adapt the training materials/curriculum such as eMULTIPAP courses that have been a success in high-income countries [33].

In the context of LMICs, there is a need for multifaceted strategies that reduce reliance on prolonged overseas training. Traditionally, training abroad has been common, but recent trends advocate for localized training initiatives. Strategies observed include face-to-face sessions, online modules, and experiential learning through research activities, with an increasing emphasis on the use of the internet and technology for program delivery. Collaboration between institutions within LMICs and high-income countries is interesting, along with engagement and actions to address ‘brain drain’ concerns. Financial support for research activities  is significant for capacity-building efforts.

Using structured frameworks, mixed-method approaches, large sample sizes, and stakeholder participation are just a few of the three research’ noteworthy qualities. Although they did not have a control group or long-term follow-up, Millar et al. successfully used a train-the-trainer model [28]. Laatikainen et al. used thorough pre- and post-intervention data, but they found problems in data collection and  unpredicted confounding variables [31]. Although Lawson et al. relied largely on self-reported surveys, they created an internationally appropriate framework based on extensive consultation [29]. It is advised to use control groups, standardize data collecting, conduct longitudinal research, and incorporate objective evaluation methods to improve these methodologies.

Capacity building models for managing MLTCs in LMICs are essential to improve the capabilities of healthcare providers and improving patient outcomes. These models focus on developing tailored training programs that address the unique challenges faced by healthcare professionals in LMICs, such as limited resources and unequal high burden of disease prevalence. Diverse training methodologies, including participatory learning and culturally relevant content, have shown to significantly improve participants’ knowledge and skills in chronic disease management. However, the literature reveals a scarcity of comprehensive studies evaluating such capacity-building initiatives, highlighting the need for ongoing support and monitoring to ensure their sustainability and effectiveness. Localized training approaches, rather than reliance on overseas programs, are increasingly advocated, utilizing technology and collaboration between institutions to enhance accessibility and relevance. Engaging the community and securing financial support for research activities can further strengthen these initiatives, equipping healthcare providers in LMICs to deliver comprehensive care for patients with multiple chronic conditions.

## Strengths and limitations

This systematic review fills a critical gap by assessing training models for managing MLTCs in LMICs. Its PRISMA adherence, PROSPERO registration, and application of a structured training framework for analysis are among its strengths. The review makes useful suggestions, such as using "train-the-trainer" approaches and incorporating MLTCs into curricula. However, generalizability was limited, because only four diverse research fulfilled the inclusion criteria. Most lacked data on patient outcomes or long-term follow-up. Due to limited geographic representation and the assessment of just English-language papers, pertinent evidence may have been disregarded. In spite of this, the review offers guidance for next capacity-building initiatives.

## Implications for policy and practice

Despite some initiatives in LMICs, they are often externally supported and short-lived, with the management of MLTCs not integrated into health curricula. As the burden of MLTCs rise in LMICs, there is a pressing need for locally relevant research to bridge the evidence-implementation gap. Policymakers should prioritize the integration of MLTCs management into healthcare training curricula and support sustained, locally driven capacity-building efforts to address the complex healthcare needs of these populations effectively.

Of particular importance is the need for MLTCs management training among healthcare professionals and students in LMICs which is an important step in strengthening primary care and advocating for policy reform in medical health education and training. Overcoming this important gap is relevant to prepare healthcare providers to effectively manage the complexities of multiple chronic conditions, improving patient outcomes and quality of care globally.

## Conclusion

This review highlights the significance of integrating a transdisciplinary, collaborative, team-based approach into health professional programs. While the existing literature provides valuable insights, there remains a gap in research, necessitating further investigation to improve our understanding of multimorbidity capacity-building. Future research efforts should involve a diverse range of stakeholders, including students, curriculum developers, academic administrators, and practitioners from various multidisciplinary health services fields.

## Supplementary Information

Supplementary file 1.

## Data Availability

All data underlying this research is available in public domain. No datasets were generated or analysed during the current study.

## Abbreviations

MLTCs: Multiple long-term conditions
LMICs: Low-and-middle income countries
HIC: High-income countries
NICE: National Institute for Health and Care Excellence
NCD: Non-communicable diseases
PRISMA: Preferred Reporting Items for Systematic Reviews and Meta-analysis
MeSH: Medical subject headings

## Declarations

All methods were carried out in accordance with the relevant guidelines and regulations.
